# Human adipose dynamics and metabolic health

**DOI:** 10.1111/nyas.12009

**Published:** 2013-01-14

**Authors:** Bin Feng, Tracy Zhang, Haiyan Xu

**Affiliations:** 1 Hallett Center for Diabetes and Endocrinology, Rhode Island Hospital, Warren Alpert Medical School of Brown UniversityProvidence, Rhode, Island; 2 Sharon High SchoolSharon, Massachusetts

**Keywords:** WAT, BAT, obesity, lipodystrophy

## Abstract

The two types of adipose tissue in humans, white and brown, have distinct developmental origins and functions. Human white adipose tissue plays a pivotal role in maintaining whole-body energy homeostasis by storing triglycerides when energy is in surplus, releasing free fatty acids as a fuel during energy shortage, and secreting adipokines that are important for regulating lipid and glucose metabolism. The size of white adipose mass needs to be kept at a proper set point. Dramatic expansion of white fat mass causes obesity—now become a global epidemic disease—and increases the risk for the development of many life-threatening diseases. The absence of white adipose tissue or abnormal white adipose tissue redistribution leads to lipodystrophy, a condition often associated with metabolic disorders. Brown adipose tissue is a thermogenic organ whose mass is inversely correlated with body mass index and age. Therapeutic approaches targeting adipose tissue have been proven to be effective in improving obesity-related metabolic disorders, and promising new therapies could be developed in the near future.

## Development of human adipose tissue

### Development of white adipose tissue

Two types of adipose tissue exist in humans, white adipose tissue (WAT) and brown adipose tissue (BAT). Abundant WAT is distributed throughout the entire body and has the capacity to expand massively. WAT is heterogeneous and contains multiple cell types, mostly white adipocytes. White adipocytes have a single large cytoplasmic lipid droplet with the nucleus located to the side of the cell. In WAT, white adipocytes are surrounded by fibroblasts, endothelial cells, immune cells, and nerves.^1^ In a human fetus, white adipose tissue appears in the six principal fat deposit sites as early as the second trimester, an important period for WAT development. In the head and neck adipogenesis begins at 14 weeks of gestation and distinct fat lobules are observed at 17 weeks. WAT is identifiable in the posterior wall of the thorax at 15 weeks and in the mammary region and anterior wall of the thorax at 16 weeks. Adipogenesis in the abdomen occurs around 14.5 weeks, and WAT development is completed along the aorta and in the pararenal fascia after 21 weeks. WAT development in the upper and lower limbs begins around 16 weeks, and the limbs are surrounded by WAT after 23 weeks of gestation.^2^

**Figure 1:**
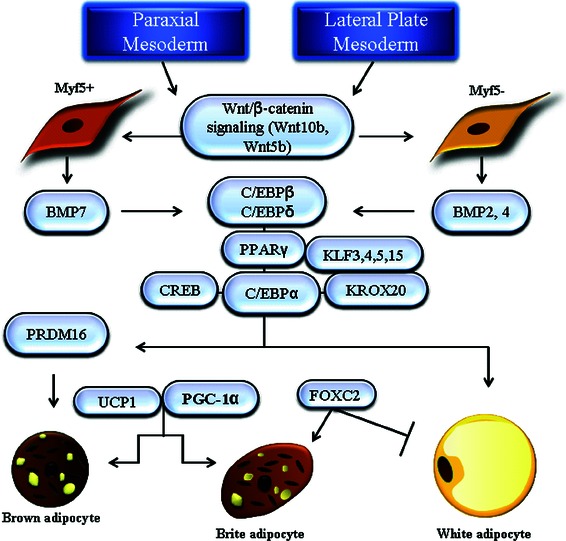
Origin and adipogenesis of white and brown adipocytes. Positive regulators involved in preadipocyte commitment and terminal differentiation of white, brite, and brown adipocytes are outlined.

White adipocytes mostly derive from the lateral plate mesoderm (Fig. 1) from stem cells that are negative for the myogenic factor Myf5.^3^ There is evidence showing that a subset of facial adipocytes originate from neural crest.^4^ WAT development begins with an accumulation of a dense mass of mesenchymal stem cells (MESCs) at various sites while angiogenesis occurs.^5^ MESCs develop into adipocytes near the networks of capillaries.^5^,^6^ Early fat cell clusters then develop into white adipose tissue, consisting of vascular structures and densely packed white adipocytes. Due to nutrition restriction, WAT development is much slower in the uterus compared to after birth. Intervention of maternal nutrition at different stage of gestation has different effects on fetal adiposity.^7^ Compromised placenta nutrient restricts the development of fetal adipose tissue, but infants will have an accelerated adipose tissue growth in early postnatal period when nutrient supply is sufficient, especially in visceral adipose depot due to increased adipogenic and lipogenic capacity of adipocytes.^8^,^9^ Both visceral and subcutaneous adipose depots are well developed at birth and continue to develop throughout adult life. Fat mass can vary anywhere from 2–3% of body weight to 60–70% of body weight.^10^ The majority of adipose tissue development is completed in early life, and fat mass expansion in later life is thought to be mainly due to enlargement of the size of white adipocytes. The number of adipocytes is relatively stable, and the turnover rate is approximately 10% per year in adult life.^11^ About half of the white adipocytes in humans are replaced every 8.3 years.^11^

### Adipogenesis of white adipocytes

Adipogenesis is a complicated process that involves the determination of preadipocytes from stem cells and the terminal differentiation of preadipocytes into mature adipocytes.^1^,^6^,^12^ It had proved difficult to differentiate adipogenic and nonadipogenic fibroblasts until recent years, when technology became available to isolate fibroblasts with adipogenic potential from the stromal vascular fraction of WAT^13^,^14^ *In vitro* studies using stem cell lines demonstrated that members of Wnt and BMP families are essential in the commitment of stem cells to preadipocytes (Fig. 1). BMP2 and 4 can stimulate dividing C3H10T1/2 stem cells to become preadipocyte-like cells, which can differentiate into adipocytes upon treatment of adipogenic stimuli after growth arrest.^15^ Wnt family members can be differential regulators of the two steps of adipogenesis: an activator of preadipocyte commitment but a repressor for terminal differentiation.^16^

In contrast to preadipocyte commitment, extensive studies have been done to understand the process of terminal differentiation (Fig. 1). Peroxisome proliferator-activated receptor γ (PPARγ), a member of the nuclear receptor superfamily, has been demonstrated to be a master regulator of adipogenesis.^6^ Two PPARγ isoforms, γ1 and γ2, exist due to alternative splicing. PPARγ2 is the isoform mainly expressed in adipose tissue and plays an important role in adipogenesis. Overexpression of PPARγ2 in fibroblasts is sufficient to induce adipocyte differentiation, but cells deficient in PPARγ lose the capability to differentiate into adipocytes.^17^,^18^ Most known adipogenic factors, such as CCAAT enhancer binding proteins (C/EBPs), cAMP response element binding protein (CREB), KROX20, and Kruppel-like factors (KLF3, 4, 5, and 15), have been shown to function through direct or indirect induction of PPARγ expression.^19^–^26^ In addition to initiation of adipogenesis in preadipocytes, PPARγ is also critical for inducing expression of a large number of genes crucial for maintaining adipocytes in a differentiated stage for proper lipogenesis and glucose metabolism.^27^ PPARγ binding sites have been identified in the regulatory regions of insulin response glucose transporter 4 (GLUT4), lipoprotein lipase (LPL), aP2, PEPCK, and adiponectin. The process of adipogenesis is also negatively regulated by antiadipogenic factors such as GATA2/3 and several members of the Wnt, hedgehog, and KLF families.^28^–^31^ Many of these adipogenesis repressors function through protein–protein interactions rather than by directly inhibiting transcription of genes critical for adipocyte differentiation.^6^

Besides protein factors, recent studies in the field demonstrate the involvement of microRNAs (miRNAs)—a family of noncoding small RNA molecules containing approximately 22 nucleotides—in regulation of adipogenesis.^32^ In mouse cell culture models, miR-17–92, miR-200, miR-103, and miR-378/378* have been identified to promote adipogenesis, whereas miR let-7, miR-27a/b, and miR-448 play roles in repressing adipogenesis. Among these mouse miRNAs, only miR-27b has been shown to inhibit adipogenesis in human preadipocytes. Additional miRNAs have also been found to regulate adipogenesis in human preadipocytes or stem cells, with miR-143 and miR-519d being proadipogenic and miR-130 and miR-138 being antiadipogenic. Several of these miRNAs exert their actions through regulating expression of known adipogenic genes such as PPARγ and C/EBPα. Further investigations in *in vivo* models are necessary to confirm and clarify the functions of these miRNAs in adipose tissue development.

### Function of white adipose tissue

WAT is the major site for storage of excessive amounts of energy in the form of triglycerides when nutrient is in surplus. During a period of energy shortage, triglycerides can be mobilized through lipolysis in order to release free fatty acids into circulation to be used by other organs as fuel. Recent studies indicate that WAT is also an active endocrine organ that secretes multiple hormones, cytokines, and chemokines important for regulating energy homeostasis. Leptin and adiponectin are two important hormones secreted by WAT. Leptin is a circulating satiety factor that suppresses food intake and increases energy expenditure.^33^ The plasma levels of leptin are positively associated with WAT mass. Adiponectin is a multimeric complement-like insulin-sensitizing hormone and one of the most abundant plasma proteins. The circulating levels of adiponectin are inversely correlated with WAT mass. Adiponectin has been considered to be a promising biomarker for indicating insulin sensitivity.^34^ WAT can also secrete proinflammatory factors such as tumor necrosis factor (TNF)-α, interleukin (IL)-6, and monocyte chemotactic protein-1 (MCP-1). Different adipose depots have distinct patterns of gene expression and adipokine secretion as well as adipogenic capacity.^35^,^36^ For example, subcutaneous adipose tissue secretes more leptin and adiponectin, but less IL-6 and free fatty acids.^37^,^38^ Human preadipocytes from subcutaneous depots have a higher capacity for adipogenesis compared with those from visceral depots.

### Development of brown adipose tissue

Brown adipocytes contain multilocular lipid droplets and are rich in mitochondria. BAT is considered to be a thermogenic organ, which expresses the uncoupling protein 1 (UCP1) and produces heat by uncoupling mitochondrial respiration from ATP synthesis.^39^ BAT is also highly vascularized and densely innervated by terminal fibers of the sympathetic nervous system.^39^,^40^ BAT is commonly found in rodents throughout their lives. In the human fetus, BAT development occurs earlier than WAT development during gestation.^41^ Brown adipocytes, along with connecting capillaries, form distinct lobules with dense amounts of connective tissue. These lobules are arranged into lobes that provide the structure of BAT.^42^ Existing evidence indicates that BAT is well developed around five months of gestation and peaks at birth.^43^ In infants, BAT can account for approximately 5% of body weight and it plays an important role in maintenance of core temperature when newborns face a sudden change from 37 °C in an intrauterine environment to a lower temperature of the external environment.^44^

**Figure 2:**
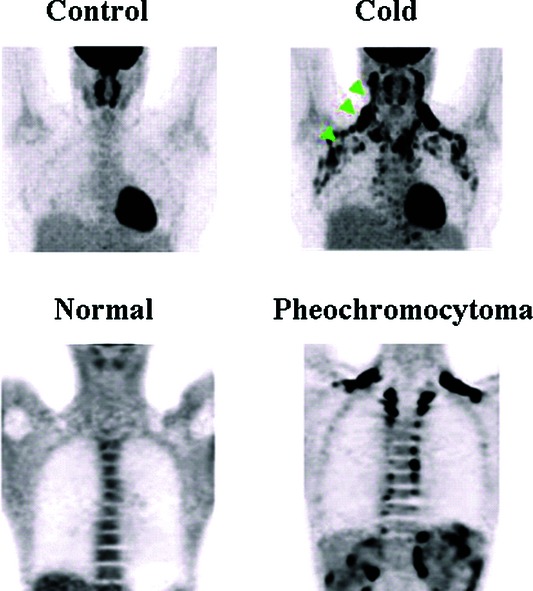
BAT activities in humans Top panels, BAT activities measured by FDG-PET in a normal subject and a subject exposed to cold (from Ref. 48). Bottom panels, BAT activities in a normal subject and a pheochromocytoma patient with elevated circulating level of catecholamines (with permission from Ref. 57).

BAT fades away in adult humans and is mostly replaced by WAT later in life. The physiological relevance of BAT in adult humans has been unclear until recent identification of metabolically active BAT in healthy adult humans, and the mass of BAT is found to be inversely correlated with age and body mass index (BMI).^45^–^47^ The result of fluorodeoxyglucose positron emission tomography (FDG-PET) indicates that BAT distributes at many sites in the adult human body, including the neck, supraclavicular depot, paravertebral depot, interscapular depot, mediastinum depot, para-aortic depot, and other depots.^48^–^51^ The supraclavicular depot is the largest BAT depot in most people.^50^ BAT has not been detected in every adult person under thermoneutral conditions, and according to a recent study, approximately 7.5% of females and 3.1% of males have detectable BAT activities under unstimulated conditions.^46^,^52^ Enhanced BAT activities are detected in adult humans under certain conditions, such as cold exposure, insulin stimulation, and elevated circulating levels of catecholamines (Fig. 2).^47^,^48^,^53^–^57^ People who work outdoors in cold temperature have more BAT mass than those who work indoors.^58^ Virtanen *et al.* found that cold induces a 15-fold increase of glucose uptake in paracervical and supraclavicular BAT, compared with a fourfold increase in WAT.^45^ They speculated that the amount of energy burnt by fully activated BAT would be equivalent to that burned by approximately 4.1 kg of WAT over the course of one year. Therefore BAT might contribute substantially to human energy expenditure. Norepinephrine has been reported to stimulate proliferation of brown preadipocytes, promote differentiation in mature brown adipocytes, and inhibit apoptosis of brown adipocytes.^39^ Patients with pheochromocytoma tend to have high concentrations of catecholamines in circulation, and activities of BAT can be detected under thermoneutral conditions.^57^ BAT activities usually diminish after tumor removal, when circulating catecholamine levels return to normal.^59^

### Classification and adipogenesis of brown adipocytes

**Figure 3:**
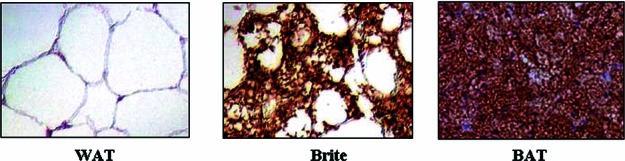
Histological images of WAT, brite cells, and BAT stained for UCP1. WAT and brite cell images are human (with permission from Ref. 45). The BAT image is mouse and from Hemmeryckx *et al.*, with permission.^201^

Brown adipocytes are also derived from the mesoderm.^3^ Two types of brown adipocytes have been identified in humans, classic interscapular-like brown adipocytes (iBAT) and inducible brown adipocytes interspersed among white fat depots in response to cold exposure or elevated plasma concentrations of catecholamine (wBAT, brite adipocytes, beige adipocytes). The comparison of histological features of WAT, brite, and BAT is shown in Figure 3. iBAT is developed from Myf5^+^ stem cells originating in the paraxial mesoderm, while brite adipocytes are derived from the lateral plate mesoderm (Fig. 1).^49^ The developmental timing is also different for these two types of brown adipocytes. In mice, iBAT is fully differentiated at birth, when fur and WAT are absent. In contrast, brite adipocytes appear around 10 days postbirth, peak around 21 days postbirth, and then regress. The disappearance of brite cells coincides with the appearance of WAT and fur. Brite cells are distinct from white and classic brown adipocytes in relation to gene expression pattern, with brite cells having low UCP1 levels in the basal state but high UCP1 levels and respiration rates in response to cyclic AMP stimulation.^60^

Brown fat depots identified in adult humans have characteristics of brite adipocytes.^60^ At a molecular level, classic brown adipogenesis involves both unique transcription factors/cofactors, such as BMP7, PRDM16, and PGC-1α, and factors that also influence WAT adipogenesis, such as PPARγ and members of the C/EBP family.^61^,^62^ PRDM16 is exclusively expressed in BAT and plays a critical role in brown preadipocyte commitment and terminal differentiation.^63^ When expressed at physiologically relevant levels, PRDM16 promotes almost all of the key features of bona fide brown adipocytes, even in the absence of PPARγ, including increased expression of PGC1α and UCP1 and enhanced mitochondrial gene expression and density. Reduction of PRDM16 expression leads to almost complete suppression of BAT-specific gene expression without affecting genes common to both BAT and WAT. PGC-1α is a cold-inducible transcription coactivator that is essential for brown adipocyte mitochondrial thermogenesis but dispensable for brown adipocyte differentiation.^64^ Ectopic expression of PGC1α in WAT promotes UCP1 expression and mitochondrion biogenesis, whereas PGC1α deficiency lowers UCP1 levels and leads to cold intolerance without affecting BAT morphology in mice.^65^,^66^ Irisin, encoded by the *Fndc5* gene, is a newly identified hormone from the muscle of PGC-1α transgenic mice.^67^ Irisin can be induced by exercise in both mice and humans. It stimulates UCP1 expression and is a potent inducer of brite cells specifically in subcutaneous WAT. Experiments in mouse models also indicate that brown adipogenesis can be suppressed by several proteins, such as RIP140, Wnt10b, and pocket proteins.^68^–^70^

## Metabolic diseases associated with changes of human adipose tissue

### Obesity

Adipose tissue is important in the maintenance of energy homeostasis. Dysfunction of adipose tissue causes various metabolic disorders. The most common adipose tissue disorder is obesity, which has now become an epidemic disease. Obesity is defined as body mass index greater or equal to 30 kg/m^2^ and features a massive expansion of white adipose tissue. Although obesity caused by genetic disorders is rare, it has been identified in humans. Patients with leptin deficiency, caused by a homozygous frame shift, nonsense, or missense mutation of the leptin gene, for example, have an undetectable level of leptin in the circulation and develop early-onset severe obesity.^71^–^73^ Metabolic disorders associated with leptin deficiency can be successfully corrected by administration of recombinant human leptin.^74^ Loss-of-function mutations in leptin receptor cause similar clinical phenotypes to those of leptin deficient patients.^75^,^76^ In other cases, mutations in melanocortin-4 receptor (MC4R), which disrupts normal expression and trafficking of MC4R to the cell surface, account for 5–6% of patients with severe early-onset obesity.^77^,^78^ And mutations in POMC, prohormone convertase 1 (PC1), tyrosine kinase receptor tropomycin-related kinase B (TrkB), brain-derived neurotrophic factor (BNDF), or single minded 1 (SIM1) have been found to cause severe obesity in humans.^79^–^85^

In addition to obesity caused by monogenic mutations, polygenic mutation–related obesity has been described in humans. For example, mutations in more than 14 genes have been identified in Bardet–Biedl syndrome, which is an autosomal recessive disease featuring obesity and other abnormalities such as learning difficulties, retinal dystrophy, renal dysfunction, and hypogonadism.^86^,^87^ Lack of gene expression due to imprinting on chromosome 15q11–13 leads to Prader–Willi syndrome, which is characterized by obesity, mental retardation, short stature, hypogonadism, and hypotonia.^88^,^89^ Deletion of a 220 kb fragment on chromosome 16p11.2, which includes the SH2B1 gene, is also found to be associated with familial severe early-onset obesity.^90^

Sedentary lifestyles and energy-dense diets are the major causes of the obesity epidemic. Humans with low birth weight are also at increased risk for developing obesity and related metabolic disorders. The limited nutrition in the uterus restricts fetal adipose tissue development in order to protect the development of vital organs, and therefore it sensitizes adipose tissue (mainly visceral adipose depot) for fat deposition in the postnatal period when nutrient supply is no longer restrained.^8^,^91^ Obesity is a risk factor for the development of many chronic diseases, such as cardiovascular diseases, hypertension, dyslipidemia, nonalcohol fatty liver disease, certain forms of cancer, and, particularly, insulin resistance and type 2 diabetes. The mass of visceral adipose depots, not subcutaneous adipose depots, is positively correlated with obesity-related metabolic disorders. Central obesity can also be caused by chronic exposure to excessive amounts of glucocorticoids, which is the common clinical feature of Cushing syndrome. The elevated levels of glucocorticoids can be either exogenous, from chronic glucocorticoid therapy, or endogenous, from pituitary adenoma, adrenal adenoma, or hyperplasia.^92^ Glucocorticoids have been reported to increase appetite and stimulate lipoprotein lipase activity, preferentially in visceral adipose depots.^93^ Activity of AMPK, the key cellular energy sensor that represses lipid synthesis, is reduced by 70% in visceral adipose tissue of patients with Cushing syndrome, providing a potential mechanism for glucocorticoid-promoted central obesity.^94^

### Adipose tissue inflammation

**Figure 4:**
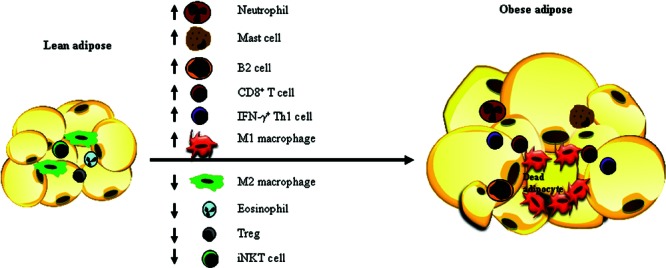
Changes of immune cell populations in adipose tissue in obesity. A Th1 response occurs in obese adipose tissue, featured with decreased populations of anti-inflammatory immune cells, including regulatory T (T_reg_) cells, M2 macrophages, eosinophils, and iNKT cells but increased populations of proinflammatory immune cells, such as neutrophils, M1 macrophages, mast cells, B2 cells, CD8^+^ T cells, and IFN-γ^+^ Th1 cells.

Extensive studies have demonstrated that obesity-related insulin resistance and type 2 diabetes are associated with inflammation in adipose tissue.^95^,^96^ In an obese state, massively expanded adipose tissue secretes a variety of inflammatory markers, cytokines, and chemokines at elevated levels. Some of these factors, such as TNF-α, IL-6, IL-1β, and MCP-1, have been reported to impair insulin signaling.^97^–^100^ Dysregulation of adipocyte lipolysis by increased expression of adipose proinflammatory cytokines contributes to systemic insulin resistance through elevated circulating FFA levels. Multiple types of proinflammatory immune cells have been identified that increase in obese adipose tissue, such as M1 macrophages, neutrophils, CD8^+^ T lymphocytes, IFN-γ^+^ Th1 cells, B2 cells, and mast cells (Fig. 4).^101^ Anti-inflammatory immune cells that normally exist in the lean adipose tissue, such as M2 macrophages, eosinophils, regulatory T (T_reg_) cells, and invariant natural killer T (iNKT) cells, are decreased in the obese adipose tissue.^101^ The accumulation of activated macrophages in adipose tissue in obesity has been shown to secrete a variety of proinflammatory cytokines and chemokines that potentially contribute to obesity-related insulin resistance.^102^,^103^ Diet-induced obese mice with decreased adipose macrophage infiltration or macrophage ablation have reduced expression of inflammatory cytokines in adipose tissue and improved systemic insulin sensitivity.^104^–^107^ Conditional depletion of proinflammatory macrophages leads to a significant decrease of inflammatory molecules in adipose tissue and rapid normalization of insulin sensitivity.^108^ Weight loss in obese subjects has also been associated with decreased macrophage infiltration and a reduction of inflammatory gene expression in adipose tissue.^109^,^110^ On the other hand, secretion of insulin-sensitizing adiponection is reduced in obese subjects.^111^

### Adipose tissue hypoxia and fibrosis

The initiation of adipose tissue inflammation in obesity is not well understood. It is noteworthy that inflammation is usually associated with pathological adipose tissue enlargement rather than healthy adipose tissue growth.^112^ Proper vascularization is needed to support adipose tissue expansion. Rapid expansion of adipose tissue in obesity is often associated with insufficient angiogenesis. Reduced capillary density and blood flow can cause poor oxygenation in expanding adipose tissue. Indeed, hypoxia has been observed in adipose tissue of obese humans and rodents, which is considered to be a potential factor for inducing adipose inflammation.^113^,^114^ In primary and cultured adipocytes, hypoxia has been shown to induce expression of many proinflammatory genes such as TNF-α, IL-1, IL-6, MCP-1, macrophage migration inhibitory factor (MIF), and matrix metalloproteinases 2 (MMP2) and 9 (MMP9).^112^,^114^ Hypoxia is also a potential contributor to the increase of adipocyte death in obesity, which is linked to the influx of proinflammatory M1 macrophages.^115^

Another important factor affecting adipose tissue expansion is the extracellular matrix (ECM) surrounding adipocytes, which mainly contains collagen. ECM components are upregulated in adipose tissue of obese humans and rodents, causing fibrosis, a key feature of adipose tissue dysfunction.^116^,^117^ Healthy adipose tissue growth involves newly differentiated small adipocytes, appropriate vascularization, and minimal induction of ECM without causing local inflammation. Pathological adipose tissue expansion mainly occurs through enlargement of existing fat cells, accompanied by insufficient vascularization, macrophage infiltration, and severe fibrosis.^112^,^116^ Interestingly, the absence of collegen VI, which is a highly enriched ECM component in adipose tissue, leads to uninhibited adipose expansion and improvement of local inflammation.^117^

### Lipodystrophy

A proper amount of adipose tissue is critical to metabolic health. Selective loss or improper distribution of body fat, defined as lipodystrophy and lipoatrophy, can cause metabolic disorders, especially insulin resistance and type 2 diabetes. The severity of metabolic disorders is determined by the extent of fat loss. Lipodystrophy can be acquired or genetic (Table 1). Among acquired lipodystrophies, it is well known that HIV patients who undergo long-term highly active antiviral therapy display HIV adipose redistribution syndrome (HARS).^118^ HARS is associated with grossly enlarged intraabdominal and neck adipose depots and depletion of subcutaneous adipose depots from the face and extremities, which causes an apple-on-a-stick shape in these patients. HARS occurs in about 50% of HIV patients, who usually also display dyslipidemia and insulin resistance.^119^ In lipoatrophic subcutaneous adipose tissue, decreased adipocyte size and apoptosis are observed, which is accompanied by reduced expression levels of adipogenic genes such as PPARγ, C/EBPα, and SREBP-1, suggesting selectively impaired adipogenesis.^120^–^122^ Expression levels of leptin and adiponectin are also reduced, which may contribute to associated insulin resistance. Mitochondrial dysfunction and local inflammation are observed in both lipoatrophic subcutaneous adipose tissue and enlarged visceral adipose tissue, excluding these changes as the mechanism for shrinking subcutaneous fat. This type of lipodystrophy is the most prevalent type among all lipodystrophies.^123^,^124^

**Table 1:** Classification of lipodystrophies

	Type	Gene	Clinical features of adipose tissue
Acquired lipodystrophies	HIV adipose tissue redistribution syndrome	N/A	Depletion of subcutaneous fat from face and extremities; enlargement of fat depots in abdominal and neck areas
	Lawrence syndrome (acquired generalized lipodystrophy)	N/A	Generalized loss of subcutaneous fat from the face, trunk, abdomen, and extremities; preserved bone marrow and retroorbital fat
	Barraquer-Simons syndrome (acquired partial lipodystrophy)	N/A	Gradual and symmetrical loss of subcutaneous fat from the face, neck, upper extremities and abdomen; preserved fat in lower extremities
Genetic lipodystrophies	Berardinelli-Seip syndrome (congenital generalized lipodystrophy)	AGPAT2	Generalized fat loss in subcutaneous, intraabdominal, intermuscular and intrathoracic regions; preserved fat in palms, soles, periarticular and retroorbital regions
		BSCL2	No body fat at birth
		Caveolin 1	Similar to AGPAT2 deficiency but has preserved bone marrow fat.
		PTRF	Similar to AGPAT2 deficiency but has preserved bone marrow fat
	Familial partial lipodystrophy	LMNA	Loss of subcutaneous fat in both extremities and the trunk
		PPARγ	Loss of subcutaneous fat in the extremities
		AKT2	Loss of subcutaneous fat in the extremities
		PLIN1	Loss of subcutaneous fat in the extremities
		CIDEC	Loss of subcutaneous fat in the extremities

Other less prevalent types of acquired lipodystrophies include Lawrence syndrome (acquired generalized lipodystrophy, AGL) and Barraquer–Simons syndrome (acquired partial lipodystrophy, APL).^125^ Both AGL and APL are early-onset lipodystrophies and affect more females than males. AGL patients suffer from generalized loss of subcutaneous fat, including the face, trunk, abdomen, and extremities, but bone marrow and retroorbital fat are unaffected. APL patients feature gradual subcutaneous fat loss starting from the face and symmetrically spreading downward to the neck, upper extremities, and abdomen, with fat in lower extremities spared. Most AGL patients have hepatic steatosis, hypertriglyceridemia, and diabetes. APL has been found to associate with several autoimmune diseases, such as systemic lupus erythematosus and dermatomyositis.

Genetic lipodystrophies are rare diseases that occur in less than one in a million people and, like acquired lipodystrophies, they are identified in females more often than in males.^126^ Berardinelli–Seip syndrome (congenital generalized lipodystrophy, CGL) patients account for about 30% of genetic lipodystrophy cases reported to date. CGL is an autosomal recessive disorder.^127^,^128^ Due to near complete absence of body fat, CGL infants are easily recognized at birth. These children have accelerated growth due to strong appetite and often develop diabetes, hyperlipidemia, hepatic steatosis, and polycystic ovaries later in life. Deficiencies of at least four genes have been identified in CGL patients: 1-acylglycerol-3-phosphate *O*-acyltransferase 2 *(AGPAT2*), Berardinelli-Seip congenital lipodystrophy 2 (BSCL2, seipin), caveolin 1 (CAV1), and polymerase I and transcript release factor (PTRF).^129^–^132^ AGPAT2 is abundantly expressed in adipose tissue, and it catalyzes synthesis of triglycerides and phospholipids by acylating fatty acid at the sn-2 position of glycerol.^133^ *BSCL2* encodes a protein named seipin, which is essential for proper lipid droplet formation and adipocyte differentiation through maintaining sustained expression of PPARγ and C-EBPα, which are critical for inducing expression of genes involved in triglyceride synthesis, such as AGPAT2, lipin1, and DGAT2.^134^,^135^ CAV1 encodes caveolin 1, a protein that resides in cell surface lipid rafts and forms microdomains termed caveolae. Caveolin 1 may mediate fatty acid transport from cell surface to lipid droplets through movement and merging of caveolae vesicles to lipid droplets. PTRF is essential for regulating proper location of caveolins and formation of caveolae.^132^ Mutations in AGPAT2 and BSCL2 are the most commonly found ones in CGL patients, while mutations in CAV1 and PTRF are rare. Among the four subtypes of CGL, mutations in BSCL2 produce the most severe phenotype, as patients are born with no body fat. Patients with the other three subtypes of CGL lose fat in subcutaneous, intraabdominal, intermuscular, and intrathoracic regions but have well-preserved adipose tissue in palms, soles, and periarticular and retroorbital regions. Patients with mutations in *CAV1* and *PTRF* also have bone marrow fat preserved.^131^,^136^,^137^

In comparison to the Berardinelli–Seip syndrome, familial partial lipodystrophy (FPL) is a less severe form of genetic lipodystrophy, characterized by fat loss in patients starting from childhood, puberty or even a later stage. Five FPL genes have been identified: LMNA (Dunnigan syndrome), PPARγ, AKT2, PLIN1, and CIDEC.^138^–^141^ LMNA encodes lamins A and C, proteins localized in the nuclear envelope, and dysfunction of lamins may cause adipocytes to die prematurely.^138^,^142^ PPARγ is a master transcription factor for adipocyte differentiation, and AKT2 is an important component of insulin signaling; the absence of either PPARγ or AKT2 impairs adipocyte development. PLIN1 encodes perilipin 1. Both perilipin 1 and CIDEC are lipid droplet-coating proteins that protect the droplets from being hydrolyzed. Recent studies indicate that perilipin 1 and CIDEC are also involved in hepatic steatosis and foam cell formation.^143^,^144^

FPL caused by mutations in LMNA, PPARγ, AKT2, and PLIN genes is autosomal dominant, while FPL caused by mutation in the CIDEC gene is autosomal recessive. Among the five subtypes of FPL, Dunnigan syndrome (more than 300 patients) is most prevalent, followed by mutations in PPARγ (30 patients), PLIN1 (6 patients), AKT2 (four patients), and CIDEC (1 patient).^126^ Dunnigan syndrome is the most severe FPL, characterized by subcutaneous fat loss in both extremities and the trunk. The remaining four subtypes of FPL involve only subcutaneous fat loss in the extremities. FPL patients usually develop diabetes and metabolic disorders in adulthood.

In addition to CGL and FPL, lipodystrophies can also be associated with other genetic diseases, such as MAD (mandibuloacral dysplasia), MDP (mandibular hypoplasia, deafness, and progeroid features), and autoinflammatory syndromes.

### Adipose tissue-derived tumors

Abnormal white adipose tissue development can produce tumors. The most common white adipose tumor is lipoma, which is a mobile, soft, and benign tumor that affects approximately 1% of the general population. Lipomas usually occur in people that are 40–60 years old but can also affect children.^145^,^146^ Lipomas are commonly observed in subcutaneous tissue between skin and muscle, though they can occur in any part of the body. Lipomas are classified into several subtypes depending on the location of the tumor, and other tissue types coexist with fat. These tumors usually do not cause any symptom and treatment is not necessary in most cases. Surgical removal is needed in some cases if the location or the size of lipomas cause pain or other problems. Lipomas may begin to grow after a tissue injury or trauma, and they tend to run in families. Human studies showed that a C-terminally truncated HMG I-C product with three DNA binding domains fused to other gene products is frequently found in lipomas. Transgenic mice overexpressing the three DNA domains of HMG I-C fused to various proteins have increased neonatal adipose tissue growth and a high incidence of lipomas, indicating that the C-terminally truncated HMG I-C protein maybe important in promoting lipoma formation.^147^

White adipose tissue can also develop into malignant liposarcoma, which mostly occurs in deep soft tissue and accounts for about 20% of all mesenchymal malignancies.^148^ Liposarcoma is divided into several subtypes based on the extent of cell differentiation and cell morphology: well-differentiated, dedifferentiated, myxoid, round cell, and pleomorphic liposarcomas. Well-differentiated liposarcoma accounts for 40–45% of liposarcomas with equal occurrence in the limbs and the retroperitoneum. This type of liposarcoma has approximately a 30% chance of local recurrence but does not metastasize. Dedifferentiated liposarcoma is characterized with a loss of lipogenic morphology in well-differentiated liposarcoma, occurring 90% of the time in the primary tumor and 10% of the time in recurrences. Dedifferentiation is accompanied by a 15–20% metastatic rate. Myxoid and round cell liposarcomas tend to occur in the limbs, and they represent 30–35% of liposarcomas. Pleomorphic sarcoma is the rarest aggressive subtype and tends to be observed in the limbs with a 30% metastatic rate.

Brown adipose tissue can also form tumors called *hibernomas*, which are rare and slow-growing benign tumors. The name derives from the morphologic similarity between the tumor and hibernating glands of animals.^149^ Hibernomas tend to occur in the thigh, shoulder, interscapular area, neck, chest, axilla, abdominal cavity, and retroperitoneum.^150^ Hibernomas most frequently occur in people in their thirties and affect females more often than males.^151^ The tumors are well encapsulated, soft, and mobile, and they are highly vascularized and composed of a mixture of brown and white adipocytes. A typical hibernoma contains more than 70% brown adipocytes—the color varying from tan to brown. Patients usually have no symptoms from the tumor itself unless its mass affects adjacent structures. It remains to be explored whether hibernomas affect energy homeostasis in humans.

## Targeting human adipose tissue for treating obesity-related metabolic disorders

### Mechanism of insulin-sensitizing thiazolidinediones

Massive expansion of white adipose tissue mass is the basis of obesity and its associated metabolic disorders. Thus, it is logical to target WAT for improving obesity-related metabolic syndromes, particularly insulin resistance and type 2 diabetes.

**Figure 5:**
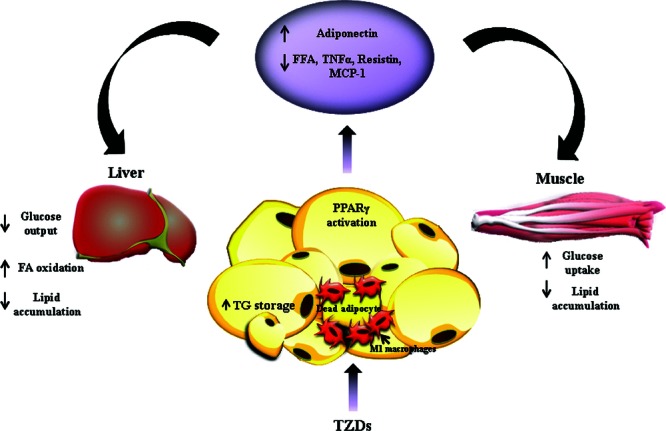
Mechanism of action for thiazolidinediones (TZDs). TZDs mainly improve adipose tissue insulin sensitivity by activating PPARγ in both adipocytes and infiltrated macrophages. Activation of PPARγ in adipose tissue promotes lipid storage in fat, decreases lipid deposit in muscle and liver through lowering circulating levels of FFA, stimulates secretion of adiponectin, and reduces secretion of proinflammatory factors, such as TNF-α, resistin, and MCP-1.

Thiazolidinediones (TZDs) are a class of insulin-sensitizing drugs that improve glycemic control of type 2 diabetic patients mainly through targeting adipose tissue (Fig. 5).^139^ Mouse models of lipodystrophy demonstrate compromised responses to TZD treatment. TZDs are direct agonist ligands for PPARγ, which is most abundantly expressed in adipose tissue.^152^ Mice with adipose tissue-specific PPARγ deletion have impaired responses to TZD treatment,^153^ and humans with dominant-negative PPARγ mutations have markedly attenuated responses to PPARγ ligands.^154^ Activation of PPARγ in adipocytes by TZD treatment enhances the lipid storage capacity of adipocytes and prevents detrimental lipid deposit in other insulin sensitive tissues, such as liver and muscle, by lowering circulating FFA levels.^27^,^155^ This effect, assumed to be achieved through promoting *de novo* differentiation of small but insulin-sensitive adipocytes, results in selective subcutaneous adipose depot accumulation.^139^,^156^ In contrast, the visceral adipose depot is either unchanged or reduced. TZD treatment also changes the secretion profile of adipocytes by enhancing production of insulin-sensitizing adiponectin, a direct target gene of PPARγ, but reducing secretion of proinflammatory factors such as TNF-α, resistin, and PAI-1.^157^ Adiponectin has been considered a candidate protein to mediate the insulin-sensitizing effects of TZDs since mice overexpressing an active form of adiponectin have similarly improved insulin sensitivity and adipose tissue redistribution to those treated with TZDs.^158^

In addition to the effects on adipocytes, TZDs also have potent anti-inflammatory effects and suppress adipose macrophage gene expression *in vitro* and *in vivo*, as well as inhibit proinflammatory mononuclear cells.^159^–^164^ In a macrophage-specific PPARγ deficient mouse model, TZD only partially improves insulin sensitivity, suggesting that the antidiabetic effect of TZD is partially attributable to PPARγ activation in macrophages.^159^ Existing evidence has demonstrated that PPARγ activation promotes alternative macrophage activation through switching phenotype from proinflammatory M1 to antiinflammatory M2.^165^ Furthermore, a unique regulatory T cell population has been found to reside in visceral adipose tissue of normal individuals and plays an important role in regulating adipose tissue inflammation and insulin sensitivity.^166^ A recent study demonstrated that the complete insulin-sensitizing effect of pioglitazone is dependent on the expression of PPARγ in visceral adipose T regulatory cells.^167^

### Alternative PPAR compounds

Despite the beneficial effects of TZDs on improving glycemic control, considerable side effects have been reported, such as body weight increase, fluid retention, hemodilution, and heart failure. Among the three potent and highly PPARγ-selective TZDs that have been extensively used in clinical practices (troglitazone, pioglitazone, and rosiglitazone), troglitazone has been taken off market because of liver toxicity. Pioglitazone and rosiglitazone are currently being used but have received black-box warnings because of increased risks for bladder cancer and cardiac attack, respectively. Extensive work has focused on the development of a new generation of PPARγ drugs that retain comparable potency to the three originally approved TZDs but with less toxicity.

A nonagonist PPARγ ligand SR1664, which blocks obesity-linked PPARγ phosphorylation by Cdk5, has been recently found to have potent antidiabetic activity without causing side effects such as fluid retention and weight gain.^168^ Additional new potential compounds include PPARγ agonists outside of the TZD class, dual agonists for PPARα/γ, PPAR pan-agonists, PPARγ antagonists, and selective PPARγ modulating agents.^169^,^170^ There is also considerable interest in exploring the possibilities of using alternative natural ligands of PPARγ for treating diabetes, such as dietary lipids or lipid derivatives, dietary isoflavones, and flavonoids.^171^

### Targeting adipose inflammation

Alternative approaches have also been explored to curb obesity-related inflammation, which originates from adipose tissue. Salsalate, a prodrug of salicylate that has been used to treat arthritis for years, has been used in clinical trials for treating obesity-related metabolic disorders since it is considered safe and well tolerated. In a small pilot study involving 20 obese nondiabetic young adults, salsalate has been shown to increase circulating levels of adiponectin and reduce glycated albumin after one month of treatment.^172^ In a later study involving 104 obese diabetic patients, salsalate treatment over a three-month period significantly improved glycemic control and lowered triglyceride levels.^173^ Mild gastrointestinal symptoms and tinnitus are observed among patients who take salsalate. The release of the results from a recently completed National Institutes of Health-funded large clinical trial will provide more information regarding the therapeutic implication of salsalate in improving obesity-related insulin resistance and type 2 diabetes.^174^ Anakinra, a recombinant human IL-1 receptor antagonist, has also been shown to effectively reduce inflammatory markers and improve insulin sensitivity in type 2 diabetic patients after 13 weeks of treatment.^175^ In contrast, antagonizing TNF-α fails to improve glycemic control in obese diabetic patients.^176^,^177^

### Other approaches targeting obesity

Other approaches targeting WAT, such as blockage of WAT angiogenesis, are still in early stages due to the lack of human trials, but data from animal experiments are promising. Inhibition of angiogenesis by angiostatin, endostatin, TNP-470, Bay129566, and thalidomide in obese mice impaired WAT expansion.^178^ Angiopoietins and their receptors, as well as the apelin-APJ signaling pathway, can also be promising targets for antiangiogenesis of WAT.^179^,^180^ Established vasculature of WAT could be targeted as well since data from animal studies showed that a cytotoxic peptide fused to the ligand of prohibitin, a receptor specifically located to WAT endothelium, resulted in rapid obesity reversal upon daily subcutaneous injection;^181^ similar results were obtained from a rat model of obesity.^182^ Future translational studies will be necessary to validate this approach.

Bariatric surgery seems the most effective approach to induce weight loss and resolve type 2 diabetes through a combination of reduction in food intake and stimulation of GLP-1 secretion.^183^ Bariatric surgery also has beneficial effects on improving cardiovascular events and mortality. However, this approach is limited to people with a BMI of either >40 kg/m^2^ or >35 kg/m^2^ and obesity-related complications. In contrast, direct removal of WAT by large volume liposuction from subcutaneous adipose tissue does not improve metabolic profile or coronary heart diseases.^184^ This is not surprising since subcutaneous adipose tissue is not the fat depot that is associated with metabolic syndrome. Omentectomy, a surgical procedure to remove the omental fat depot, does not have additional benefits in patients undergoing bariatric surgery.^185^ The beneficial effect on metabolism from bariatric surgery is so dramatic that it is likely to mask any smaller effect generated by omentectomy. Additional randomized trials involving omentectomy alone without bariatric surgery will be necessary to clarify the importance of the omental fat depot in obesity-related metabolic disorders.

### Targeting BAT for reducing adiposity

The transient weight loss achieved through dietary regimens and/or exercise is often regained when people fail to be persistent. It has now been recognized that a slow metabolism can develop in response to reduced food intake and body weight loss. This is reflected by a lower rate of resting energy expenditure, enhanced metabolic efficiency, and decreased capacity of the body to burn fat via oxidation, leading to diminished thermogenesis.^186^ Approaches that can counteract the energy-sparing mechanism will be appealing in obesity management. Activation of mitochondria uncoupling is a potential way to increase energy expenditure by producing heat. Dinitrophenol was developed and marketed to promote thermogenesis. Yet, despite a marked effect on body weight reduction, dinitrophenol was withdrawn due to unbearable side effects such as frequent sweating and hyperthermia.^187^,^188^ Thyroid hormone is also very efficient in increasing energy expenditure, but its use in treating obesity carries a high risk of cardiac stimulation and increased protein catabolism.^189^ Suppression of the sympathetic nervous system (SNS) has been recognized as the major mechanism in calorie restriction-induced reduction of thermogenesis.^190^ Clinically relevant doses of sympathomimetic drugs such as caffeine and ephedrine have thermogenic effects in both lean and obese humans.^191^,^192^ However, there was little enthusiasm to market these drugs due to side effects such as hypertension, tremor, and tachycardia. The identification of β3-adrenergic receptor in brown adipocytes kindled new hope since this receptor is an important mediator of SNS-generated thermogenesis and fat oxidation.^193^ In rodents, ablation of BAT and all β-adrenergic receptors causes obesity, and transgenic mice overexpressing UCP1 are protected from diet-induced obesity. Selective agonists of β3-adrenergic receptor were expected to lack cardiovascular side effects. Indeed, β3-adrenergic receptor agonists have potent antiobesity effects in rodents without the side effects commonly observed in general β-adrenergic receptor agonists.^194^ However, the antiobesity effect is too mild in humans to generate a marketable antiobesity drug.^194^

The recent discovery of functional BAT in humans revitalized interest in targeting this nonshivering thermogenic tissue for treating obesity. It has been estimated that activation of approximately 50 g of BAT is sufficient to increase by about 20% the daily energy expenditure in a person expending 2,500 kcal per day, which is enough to prevent body weight from rebounding after diet and/or exercise-induced weight loss.^189^,^195^ Alternative BAT-activating drugs can be developed to lower set-point of body weight. Recent advances in stem cell technology make BAT transplantation a potentially attractive therapy for enhancing energy expenditure.^196^ Human pluripotent stem cells successfully differentiated into mature brown adipocytes *in vitro* have been shown to develop into functional brown adipose tissue in an immunocompromised mouse model.^197^ BAT transplantation has also been shown to reverse type 1 diabetes in both immune-competent and immune-deficient streptozotocin-treated mice, indicating a possibility of restoration of euglycemia in mouse models of type 2 diabetes.^198^ More experiments will be needed to evaluate whether BAT transplantation is sufficient to reduce adiposity, enhance insulin sensitivity, and improve glycemic control.

In addition to the development of BAT-activating drugs and BAT transplantation, a biotherapeutic approach is feasible, which consists of inducing BAT formation in white adipose tissue. The recently identified muscle derived hormone irisin is a potential candidate. Overexpression of irisin in the liver of mice, via adenoviral delivery, increases energy expenditure and reduces high fat diet-induced obesity.^67^ These data indicate that irisin could have the potential to induce WAT browning—the induction of brite cells in WAT—in human subcutaneous adipose tissue. Despite the fact that the FNDC5 gene is only induced in older adults by exercise and seems unrelated to metabolic status of the majority of subjects, circulating irisin levels have been positively correlated with bicep circumference^199^,^200^ and are several fold higher in young male athletes than in middle-aged obese women.^200^ The effect of irisin on inducing brite adipocytes in human WAT remains to be investigated. If proven successful in human trials, irisin may be a promising antiobesity agent.

## Conclusions

Considerable work on adipocyte biology during the past decade has demonstrated the importance of adipocytes in a variety of physiological processes. WAT has been redefined as an active endocrine organ rather than a passive storage site for lipids. BAT, previously considered physiologically irrelevant in adult humans, has been rediscovered in various regions of the adult human body and demonstrated to be active in metabolism. The obesity epidemic has increased the incidence of many diseases, such as type 2 diabetes, heart diseases, and certain forms of cancer; it also poses a huge financial burden to society. In the United States alone obesity-related metabolic disorders are estimated to cost $147 billion annually.

The two currently available antiobesity drugs, orlistat and phentermine, which block intestinal fat absorption by inhibiting pancreatic lipase and repress appetite, respectively, are not very effective. The FDA recently approved a third obesity drug, lorcaserin, a selective 5-HT_2C_ receptor agonist, that induces satiety; however, lorcaserin is expected to modestly benefit only people who diet and exercise. New effective therapeutic agents are urgently needed, and adipose tissue could be the right target. Approaches aiming to reduce adipose lipid content will have to consider where instead to deposit lipids in order not to burden other tissues or organs. Systemic lipotoxicity has been demonstrated to cause a variety of problems, such as hepatosteatosis, β-cell damage, and cardiovascular diseases. Stimulation of lipid oxidation or repression of appetite, in combination with agents blocking adipose tissue lipid storage, would seem to be ideal treatment outcomes for obesity.
